# Target and non-target vessel related events at 10 years post percutaneous coronary intervention

**DOI:** 10.1007/s00392-022-01986-4

**Published:** 2022-02-11

**Authors:** J. J. Coughlan, Alp Aytekin, Erion Xhepa, Salvatore Cassese, Michael Joner, Tobias Koch, Jens Wiebe, Tobias Lenz, Tobias Rheude, Constanza Pellegrini, Senta Gewalt, Tareq Ibrahim, Karl-Ludwig Laugwitz, Heribert Schunkert, Adnan Kastrati, Sebastian Kufner

**Affiliations:** 1 Deutsches Herzzentrum München, Technische Universität München, Lazarettstrasse, 36, Munich, Germany; 2 Klinik Und Poliklinik Innere Medizin I (Kardiologie, Angiologie Und Pneumologie), Klinikum rechts der Isar, Technische Universität München, Munich, Germany; 3 DZHK (German Center for Cardiovascular Research), Partner Site Munich Heart Alliance, Munich, Germany

**Keywords:** Percutaneous coronary intervention, Drug eluting stents, Cardiovascular outcomes, Target vessel, Atherosclerotic coronary artery disease

## Abstract

**Aims:**

To define the incidence of events related to the stented vessel (target vessel related events: TVRE) and events related to non-stented vessels (non-target vessel related events: NTVRE) through to 10-year follow-up in patients post-PCI with newer generation drug eluting stents (DES).

**Methods and results:**

The current study is a post-hoc analysis of patient level data from two randomised controlled trials in Germany. Patients older than 18 years with ischemic symptoms or evidence of myocardial ischemia in the presence of ≥ 50% de novo stenosis located in the native coronary vessels were considered eligible. The endpoints of interest were TVRE (a composite of first target vessel myocardial infarction or target vessel revascularization) and NTVRE (a composite of first non-target vessel MI or non-target vessel revascularization) through to 10 years post PCI.

We included 4953 patients in this analysis. Through to 10-years post-PCI, TVRE occurred in 1238 of 4953 patients (cumulative incidence: 25.8%) and NTVRE occurred in 1442 of 4953 patients (cumulative incidence: 30.3%). The majority of TVRE and NTVRE were revascularization events. From 0 to 1 years, the cumulative incidence of TVRE was 15.9% and of NTVRE was 12.3%. From 1 to 10 years, the cumulative incidences of TVRE and NTVRE were 11.2% and 22.4%, respectively.

**Conclusion:**

At 10-year post-PCI with new generation drug eluting stents, events related to remote vessel disease progression account for a higher proportion of events than events related to the stented vessel.

**Trial registration:**

ISAR TEST 4 ClinicalTrials.gov Identifier: NCT00598676. ISAR TEST 5 ClinicalTrials.gov Identifier: NCT00598533.

**Graphical abstract:**

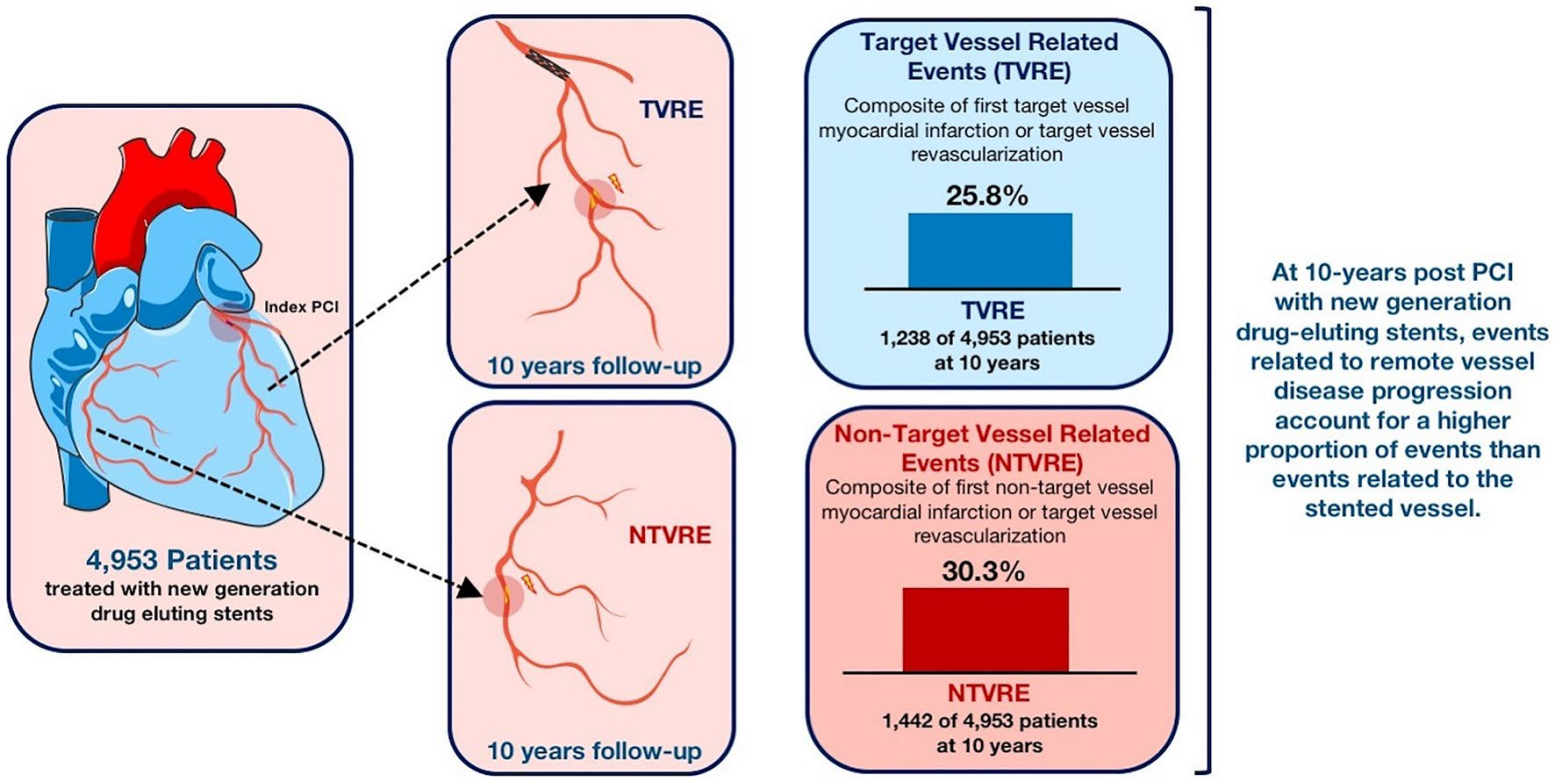

**Supplementary Information:**

The online version contains supplementary material available at 10.1007/s00392-022-01986-4.

## Introduction

Patients who undergo percutaneous coronary intervention (PCI) are at risk of subsequent cardiac events, including myocardial infarction (MI) and repeat revascularisation [1, 2]. Coronary stent trials traditionally focus on target vessel/lesion related clinical outcomes in the shorter term with a particular emphasis on outcomes in the first 12-months post-PCI [3].

It is important to consider that patients who undergo PCI are at risk of not only events related to the stented vessel (target vessel related events: TVRE) but also events related to non-stented vessels (non-target vessel related events: NTVRE) [1, 2]. While data have been published on the incidence of these event types up to 5-years post-PCI [1, 2, 4–7], their incidence at longer term follow-up has not been defined.

An analysis of TVRE and NTVRE through to 10 years after PCI in a large number of patients treated with new generation DES may be useful and help guide strategies to improve cardiovascular outcomes after PCI. Therefore, we conducted the current analysis, with the primary aim of defining the cumulative incidence of TVRE and NTVRE through to 10 years post PCI in patients enrolled in the ISAR-TEST 4 and ISAR-TEST 5 trials.

## Methods

### Study population, study device description and enrolment criteria

The current study is a pooled analysis of patient level data from the Intracoronary Stenting and Angiographic Results: Test Efficacy of 3 Limus-Eluting Stents (ISAR-TEST 4) and Intracoronary Stenting and Angiographic Results: Test Efficacy of Sirolimus- and Probucol-Eluting versus Zotarolimus-Eluting Stents (ISAR-TEST 5) randomised trials. The complete trial designs and 10-year clinical outcomes of both trials have been previously reported [8–11]. The ISAR-TEST 4 trial compared three different treatment arms in 2,603 patients. These were: biodegradable polymer based sirolimus eluting stents (BP-DES), (Yukon Choice PC, Translumina, Hechingen, Germany and Translumina Therapeutics, Dehradoon, India) (*N *= 1299), new generation permanent polymer everolimus eluting stents (PP-EES), (Xience, Abbott Vascular, Abbott Park, IL, USA), (*N* = 652) and early generation permanent polymer sirolimus eluting stents, (Cypher, Cordis Corporation, Miami Lakes, FL, USA), (*N* = 652). The ISAR-TEST 5 trial enrolled 3002 patients and compared clinical outcomes between new generation polymer free sirolimus and probucol-eluting stents (PF-DES) (*N* = 2002) and new generation permanent polymer-zotarolimus-eluting stents (PP-ZES) (Resolute, Medtronic Cardiovascular, Santa Rosa, California) (*N* = 1000). The polymer-free sirolimus- and probucol-eluting stents consist of a pre-mounted, sand-blasted, 316L stainless steel microporous thin strut (87-mm) stent, which is coated with a mixture of sirolimus, probucol, and shellac resin (a biocompatible resin widely used in the coating of medical tablets). This coating strategy is currently available in two devices from German manufacturers (ISAR VIVO, Translumina, Hechingen, Germany and Coroflex ISAR, B. Braun Melsungen, Berlin, Germany).

The current analysis includes all patients from the ISAR-TEST 4 and ISAR-TEST 5 trials, with the exception of the early generation permanent polymer sirolimus eluting stent group from ISAR-TEST 4. This group of patients were excluded from this analysis as early generation DES have been associated with a higher risk of adverse events compared to newer generation DES and their use was not felt to reflect current practice or be relevant to the study question, which is focused on new generation DES [12]. This study conforms to the declaration of Helsinki and the study protocol was approved by the ethics committee of the two participating centers in Munich, Germany (Deutsches Herzzentrum München and 1.Medizinische Klinik, Klinikum rechts der ISAR).

Enrolment criteria for both studies have been previously reported [8, 9]. Patients older than 18 years with ischemic symptoms or evidence of myocardial ischemia in the presence of ≥ 50% de novo stenosis located in the native coronary vessels were considered eligible. Of note, patients with a target lesion in the left main stem or in cardiogenic shock were considered ineligible for both studies. Relook angiography was recommended (but not mandated) by the trial protocols at 6–8-months after the index PCI.

### Endpoints and definitions

Detailed descriptions of study endpoints for both trials have been published previously [10, 11]. The primary endpoints assessed for the current analysis were TVRE (a composite of first target vessel MI (TVMI) or target vessel revascularization (TVR)) and NTVRE (a composite of first non-target vessel MI (NTVMI) or non-target vessel revascularization (NTVR)). The target vessel was defined in this analysis as the vessel/vessels treated during the index PCI. All other vessels were classified as ‘non-target vessels’. Therefore, patients could experience no events, TVRE, NTVRE or Both Events (TVRE and NTVRE) during follow-up.

Secondary endpoints included the individual components of the primary endpoints. We also analysed the median time to event for patients experiencing an endpoint through to 10 years post PCI and the interval between events for patients who experienced both a TVRE and NTVRE through to 10 years.

### Statistical analysis

Continuous data are presented as means and standard deviation (± SD) or medians and interquartile ranges. Categorical data are presented as counts and proportions. Differences between groups were checked for significance using an analysis of variance test (ANOVA) for continuous data. Chi-squared test (χ2) was used to check for differences between categorical variables. Survival was analyzed by the Kaplan Meier method to estimate the time to first event for each endpoint of interest. Hazard ratios were calculated using a Cox proportional hazards model after checking for fulfilment of the proportional hazards assumption as per the method of Grambsch and Therneau. The analysis of all endpoints accounted for the competing risk of death. In addition to the primary analysis, the cumulative incidences of TVRE and NTVRE were analysed as per stent polymer type, dividing patients into three groups; BP-DES, PP-DES and PF-DES. Statistical analysis was performed using the R 3.6.0 Statistical Package (The R Foundation for Statistical Computing, Vienna, Austria). A two tailed *p* value of < 0.05 was taken to confer statistical significance.

## Results

### Study population

We included 4,953 patients in this analysis. This represents 88.4% of the total cohort enrolled in the ISAR-TEST 4 and ISAR-TEST 5 trials. Of these patients, 1,299 (26.2%) were treated with BP-DES, 2,002 (40.4%) with PF-DES and 1,652 (33.4%) with PP-DES. Of these patients, 2,098 (42%) had a clinical event (TVRE and/or NTVRE) through to 10 years follow-up. Amongst patients with an event (*N* = 2098), 31.3% (*N* = 656) experienced a TVRE, 41% (*N* = 860) a NTVRE and 27.7% (*N* = 582) both a TVRE and NTVRE through to 10 years post PCI. Death occurred in 1,533 of 4,953 patients (31.0%) during follow-up.

### Baseline characteristics

Baseline characteristics for the patients included in this analysis as per the event type experienced are demonstrated in Table 1. Female sex was more common in patients who experienced no events. Patients who experienced no events also tended to be older at index PCI, less commonly diabetic and had a lower frequency of triple vessel coronary artery disease. Trial protocol recommended angiography (recommended to be performed at 6–8 months after PCI) was performed less frequently in the no-events group.

**Table 1:** Baseline Characteristics as per the event type experienced during 10-year follow-up

	No event	TVRE	NTVRE	Both events	*P* value
Patients	(*N* = 2855)	(*N* = 656)	(*N* = 860)	(*N* = 582)	
Age (years)	68.0 ± 11.3	66.4 ± 10.4	66.4 ± 10.3	66.5 ± 10.4	< 0.001
Female	757 (26.5)	145 (22.1)	156 (18.1)	115 (19.8)	< 0.001
Diabetes mellitus	746 (26.1)	221 (33.7)	267 (31.0)	196 (33.7)	< 0.001
*Insulin-dependent*	244 (8.6)	84 (12.8)	72 (8.4)	74 (12.7)	< 0.001
Hypertension	1883 (66.0)	459 (70.0)	587 (68.3)	412 (70.8)	0.046
Current smoker	472 (16.5)	108 (16.5)	153 (17.8)	93 (16.0)	0.793
Hypercholesterolemia	1811 (63.4)	433 (66.0)	542 (63.0)	412 (70.8)	0.005
Body mass index (kg/m^2^) *	27.4 ± 4.5	27.8 ± 4.7	27.9 ± 4.3	27.8 ± 4.0	0.007
Prior myocardial infarction	797 (27.9)	192 (29.3)	260 (30.2)	199 (34.2)	0.021
Prior aortocoronary bypass surgery	228 (8.0)	67 (10.2)	110 (12.8)	77 (13.2)	< 0.001
Number of diseased coronary vessels					< 0.001
*One vessel*	568 (19.9)	102 (15.5)	60 (7.0)	29 (5.0)	
*Two vessels*	843 (29.5)	150 (22.9)	199 (23.1)	118 (20.3)	
*Three vessels*	1444 (50.6)	404 (61.6)	601 (69.9)	435 (74.7)	
Number of lesions	1.4 ± 0.6	1.5 ± 0.7	1.4 ± 0.6	1.6 ± 0.8	< 0.001
Clinical presentation					0.022
*Acute myocardial infarction*	542 (19.0)	111 (16.9)	149 (17.3)	102 (17.5)	
*Unstable angina*	613 (21.5)	173 (26.4)	194 (22.6)	158 (27.1)	
*Stable angina*	1700 (59.2)	372 (56.7)	517 (60.1)	322 (55.3)	
Ejection fraction (%) *	52.8 ± 12.0	53.3 ± 10.7	52.5 ± 11.5	52.5 ± 10.8	0.651
Relook Angiography Performed (As Recommended by Trial Protocols)	1940 (68.0)	552 (84.1)	771 (89.7)	543 (93.3)	< 0.001

Data are mean ± standard deviation or counts (%)

*Missing continuous data:

Body-mass index was not available in 6 patients (5 in the no event group and 1 in the both events group); ejection fraction was not available in 650 patients (356 in the no event group, 85 in the TVRE group, 116 in the NTVRE group and 93 in the both events group). The remaining continuous data were complete

*TVRE* target vessel related events, *NTVRE* non-target vessel related events

### Angiographic and procedural characteristics

The angiographic and procedural characteristics for the patients included in this analysis as per the event type experienced are summarized in Table 2. Patients who experienced no events had a lower proportion of complex lesions treated during the index procedure, as well as a shorter lesion and total stented length. The left anterior descending artery was more commonly treated in the group of patients who experienced no events.

**Table 2:** Angiographic and Procedural Characteristics as per the event type experienced during 10-year follow-up

	No event	TVRE	NTVRE	Both events	*P* value
Lesions	(*N* = 3869)	(*N* = 970)	(*N* = 1173)	(*N* = 912)	
Stent polymer type					< 0.001
*Permanent polymer*	1290 (33.3)	309 (31.9)	411 (35.0)	319 (35.0)	
*Biodegradable Polymer*	964 (24.9)	276 (28.5)	295 (25.1)	148 (16.2)	
*Polymer-free*	1615 (41.7)	385 (39.7)	467 (39.8)	445 (48.8)	
Target vessel					< 0.001
*Left anterior descending artery*	1855 (47.9)	398 (41.0)	483 (41.2)	370 (40.6)	
*Left circumflex artery*	925 (23.9)	231 (23.8)	320 (27.3)	298 (32.7)	
*Right coronary artery*	1089 (28.1)	341 (35.2)	370 (31.5)	244 (26.8)	
Chronic total occlusion	205 (5.3)	49 (5.1)	54 (4.6)	67 (7.4)	0.037
Complex morphology (B2/C)	2744 (70.9)	741 (76.4)	878 (74.9)	718 (78.7)	< 0.001
Lesion length (mm)	15.7 ± 9.2	16.1 ± 10.0	16.2 ± 9.4	16.8 ± 10.0	0.005
Vessel size (mm)	2.8 ± 0.5	2.8 ± 0.5	2.8 ± 0.5	2.7 ± 0.5	< 0.001
Total stented length (mm)	24.9 ± 11.6	26.5 ± 12.3	25.8 ± 12.0	26.7 ± 12.6	< 0.001
Percent stenosis, pre-procedure (%)	66.7 ± 16.2	66.9 ± 16.2	66.4 ± 15.8	67.4 ± 15.5	0.547
Percent stenosis, post-procedure (%)	11.4 ± 7.3	12.4 ± 8.3	12.0 ± 7.4	12.4 ± 6.9	< 0.001
Balloon diameter (mm)	3.1 ± 0.5	3.0 ± 0.5	3.1 ± 0.5	3.0 ± 0.5	< 0.001
Balloon: Vessel Ratio	0.41 (0.54)	0.46 (0.55)	0.44 (0.55)	0.27 (0.49)	< 0.001
Maximal Balloon Pressure (mmHg)	15.4 (3.1)	15.6 (3.2)	15.5 (3.1)	15.8 (3.3)	0.02

Data are mean ± standard deviation or counts (%)

*TVRE*  target vessel related events, *NTVRE*  non-target vessel related events

### Primary endpoint: cumulative incidence of TVRE and NTVRE through to 10 years post PCI

**Fig. 1:**
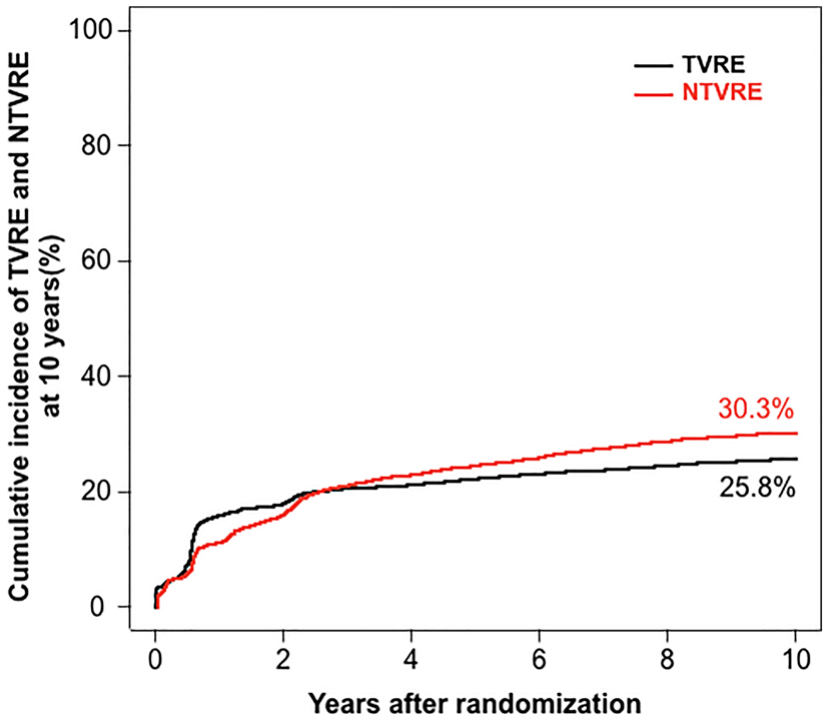
Cumulative incidence of target vessel and non-target vessel related events through to 10 years post PCI

Through to 10-years post-PCI, a TVRE occurred in 1,238 of 4,953 patients (cumulative incidence: 25.8%) and a NTVRE occurred in 1,442 of 4,953 patients (30.3%). These data are demonstrated in Fig. 1 and Table 3. The cumulative incidence of TVRE, NTVRE and ‘Both Events’ are shown in Supplemental Fig. 1.

**Table 3:** Target vessel and non-target vessel related events through to 10-years  post PCI

Total patients (*N*)	4953	
Endpoint	Number of Events	Cumulative incidence (%)
*Primary endpoint*		
Target vessel related events	1238	25.8
Non-target vessel related events	1442	30.3
*Secondary endpoint: myocardial infarction*		
Target vessel myocardial infarction	210	4.4
Non-target vessel myocardial infarction	78	1.7
*Secondary endpoint: revascularisation*		
Target vessel revascularisation	1123	23.4
Non-target vessel revascularisation	1403	29.5

### Landmark analysis

**Fig. 2:**
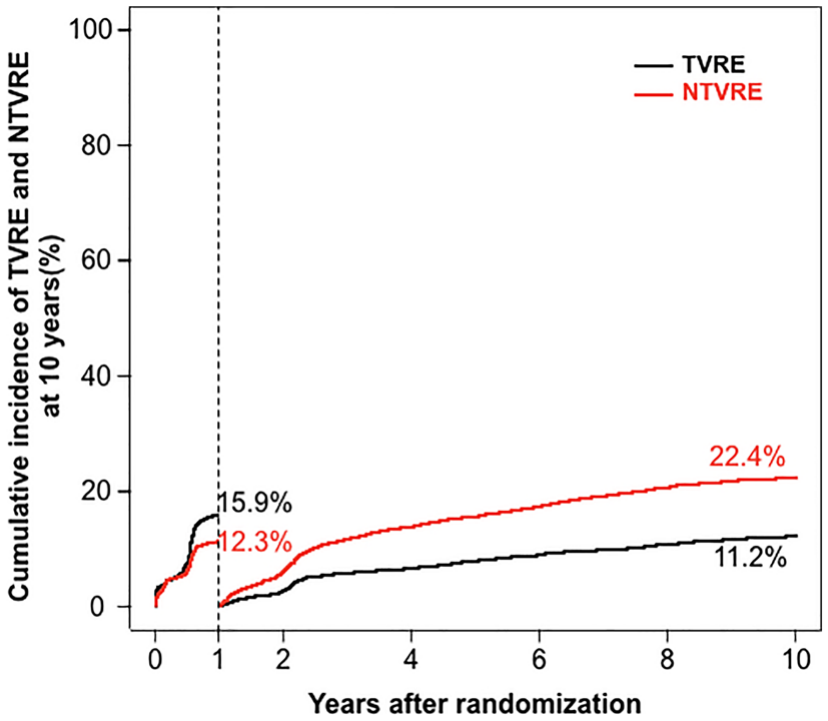
Landmark analysis of target vessel and non-target vessel related events from 0–1 and 1–10 years post PCI

A landmark analysis was then performed to assess the cumulative incidence of TVRE and NTVRE from 0 to 1 years and 1–10 years post PCI. From 0 to 1 years, the cumulative incidence of TVRE was 15.9% and of NTVRE was 12.3%. From 1 to 10 years, the cumulative incidences of TVRE and NTVRE were 11.2% and 22.4%, respectively. These data are demonstrated in Fig. 2.

### Primary endpoint: As per stent polymer technology

The frequency of both TVRE and NTVRE through 10 years was comparable for patients treated with PP-DES, BP-DES and PF-DES. This is shown in Supplemental Figs. 2, 3.

### Secondary endpoints: TV/NTV MI and TV/NTV revascularisation through to 10 years post PCI

TVMI occurred in 210 of 4,953 patients through to 10 years post PCI (4.4%) and NTVMI occurred in 78 of 4,953 patients (1.7%). TVR occurred in 1,123 of 4953 patients through to 10 years (23.4%) and NTVR occurred in 1,403 of 4,953 patients (29.5%). These data are summarised in Table 3.

### Cardiac death

Cardiac Death was identified in 138 of 656 patients (21.1.%) with isolated TVRE, 129 of 860 patients (15%) with isolated NTVRE and 102 of 582 patients (17.5%) with both events.

### Median time to first event

The median time to first event was 205 days [inter-quartile range: 132, 468] for patients with a TVRE, 684 days [193, 1369] for patients with a NTVRE and 222 days [148, 773] for patients with Both Events (*p* < 0.001).

### Time intervals between TVRE and NTVRE

For patients who experienced both TVRE and NTVRE during follow-up (*N* = 582), 205 of 582 patients had a TVRE before a NTVRE and 208 patients had a NTVRE before a TVRE. In the remaining 169 patients, both events (TVRE and NTVRE) occurred the same day. A histogram of the time intervals between TVRE and NTVRE is demonstrated in Supplemental Fig. 4. For the majority of patients, the time interval between the two event types was less than 1 year.

## Discussion

In this analysis of TVRE and NTVRE through to 10-years post-PCI with new generation DES, the main findings are as follows:

1. NTVRE account for a higher proportion of total events than TVRE at 10-year follow-up.
2. TVRE and NTVRE demonstrate distinct temporal patterns, with a higher incidence of TVRE in the first year post-PCI, followed by a higher incidence of NTVRE from year 1 to year 10.
3. Analysis of the median time to first event echoed these results, with a shorter time to first TVRE compared to first NTVRE.
4. For the majority of patients who experience both a TVRE and a NTVRE, the time interval between these events is less than 1 year.

The current report presents the only analysis of TVRE and NTVRE through to 10-years post-PCI. It also defines for the first time the incidence of TVRE and NTVRE in patients undergoing PCI with all three newer generation DES polymer technologies (BP, PP, PF) used in current practice.

The findings of this analysis build on the sometimes divergent results of several other studies reporting on events related to stented and remote vessels/lesions post-PCI [1, 2, 4, 5, 13]. Cutlip et al. demonstrated that in patients receiving PCI with second generation, bare metal coronary stents, outcomes at 5 years were determined by a high rate of events related to disease progression in segments other than the stented lesion [4]. In contrast to our study, this analysis divided events into target lesion events (TLE) and non-target lesion events (NTLE). Subsequently, Stone et al. reported that in patients with ACS undergoing PCI, major adverse cardiovascular events were equally attributable to TLE and NTLE at a median follow-up of 3.4 years [1]. This study also reported that although lesions responsible for TLE were frequently angiographically mild, intravascular ultrasonography (IVUS) analysis demonstrated a combination of characteristics in these lesions, including thin-cap fibroatheroma, large plaque burden and small luminal area. In contrast to our current analysis, where all patients were treated with newer generation DES, early generation DES were implanted in only 66% of lesions in this study. Zellweger et al. subsequently reported that in an analysis of patients from 7- to 60-months post-PCI, remote events accounted for 37.1% of all non-fatal events in a patient population treated with both bare metal stents (BMS) and early generation DES.

With regard to newer generation DES, Abdel-Wahab et al. described the incidence of non-target lesion revascularisation (NTLR) at 3-year follow-up in a large cohort of patients from the RESOLUTE global clinical trial program [7]. They reported that the cumulative incidence of NTLR was almost three times more frequent than TLR at 3-years post-PCI. However, this differed to our analysis in that it focused only on TLR/NTLR and excluded patients who had both events (TLR and NTLR). In addition, the patient cohort analysed was derived from a combination of randomised controlled trial and registry data rather than exclusively from randomised controlled trial data [7].

The timing of events in our study merits further discussion. As demonstrated in the median time to first event analysis, TVRE tend to occur earlier post-PCI than NTVRE. Accordingly, TVRE accounted for a greater proportion of events than NTVRE from in the first year post-PCI. However, after the first year NTVRE began to predominate and at 10-year follow-up NTVRE account for a higher proportion of total events than TVRE. This is similar to 5-year results previously reported by Cutlip et al. [4]. It is also notable in our analysis that, after undergoing PCI to a target vessel, over one quarter of patients will have a further event related to this vessel at long term follow-up. Given the classification of events in this analysis as target vessel rather than target lesion related, these events could have been related to either the previously implanted stent or to disease progression in remote regions of the vessel that were not treated at the index PCI. Notwithstanding this limitation, it is evident from our data that both TVRE and NTVRE contribute importantly to overall event rates at long term follow-up.

Whether novel stent polymer technologies (BP, PF) influence the proportion of TVRE and NTVRE at follow-up has not been previously studied. This is important to define, because concern has been raised regarding chronic vascular inflammation with PP-DES secondary to the presence of a permanent polymer in the vessel wall [14–17]. In addition, it has been shown in animal models that focal vascular inflammation may accelerate atherosclerotic disease in remote arterial segments [18]. Therefore, it may be logical to consider that different polymer technologies could theoretically demonstrate different frequencies of non-target vessel disease progression at long term follow-up. However, in our study, the frequency of both TVRE and NTVRE at 10 years were comparable between the three studied stent polymer technologies (PP, BP, PF).

The current analysis may also serve to highlight the importance of instituting long term secondary prevention measures after PCI to improve outcomes and challenges a solely stenting/stenosis focused interventional paradigm [19, 20]. Given that the incidence of NTVRE is unlikely to improve with iterative improvements in stent technology, novel strategies may be required, to improve long term outcomes for patients in this regard.

### Limitations

The major limitation of this analysis is that it is a post-hoc analysis of pooled data from two randomised controlled trials. This is subject to the usual limitations associated with post-hoc analysis and the findings should be regarded as hypothesis generating. Due to the inability to accurately ascribe mortality to TVRE or NTVRE, we were not able to include mortality or cardiac mortality in our primary endpoint. Planned angiographic follow-up at 6–8-months after PCI was recommended as part of the study protocols and this may have artificially altered the incidence of TVRE and NTVRE in comparison to standard clinical practice. *In detail, 6–8-month follow-up angiography was performed in 68% of the patients in the no-events group, 84.1% of patients in the TVRE group, 89.7% of patients in the NTVRE group and 93.3% of patients in the both events group *(Table 1). Intracoronary imaging or coronary physiological testing was not performed as part of the study protocols to the target or non-target vessels, and therefore, we have no information on intracoronary imaging or physiology predictors of TVRE and NTVRE. This analysis considered only the first event for both TVRE and NTVRE, and therefore, recurrent events were not accounted for. Information as to whether the procedure was staged or not was also not collected in the trial database. A final limitation of this analysis is the absence of data on rates of dual anti-platelet therapy and cardiovascular secondary prevention measures up to 10 years, both of which may have impacted on the incidence of long-term events.

## Conclusions

At 10-year follow-up post-PCI, events related to non-target vessels account for a higher proportion of total events than events related to the target vessel.

## Supplementary Information

Below is the link to the electronic supplementary material.

Supplementary file4 (JPG 43 KB) Supplemental Figure 1. Cumulative incidence of target vessel related events, non target vessel related events and both events through 10 years.

Supplementary file4 (JPG 68 KB) Supplemental Figure 2. Cumulative incidence of target vessel related events based on stent type through 10 years.

Supplementary file4 (JPG 75 KB) Supplemental Figure 3. Cumulative incidence of non-target vessel related events based on stent type through 10 years.

Supplementary file4 (TIFF 3073 KB) Supplemental Fig. 4. Histogram of the time interval between events for patients who experienced both a target vessel and non-target vessel related event through 10 years. Patients could experience a TVRE before a NTVRE, a NTVRE before a TVRE or both events on the same day

## Abbreviations

PCI: Percutaneous coronary intervention
MI: Myocardial infarction
MACE: Major adverse cardiac event
TVRE: Target vessel related event
NTVRE: Non-target vessel related event
DES: Drug eluting stent
BP: Biodegradable polymer
PP: Permanent polymer
PF: Polymer free
TVR: Target vessel revascularisation
