# Supplementary figures and images for: Target and non-target vessel related events at 10 years post percutaneous coronary intervention
Source: Clin Res Cardiol. 2022 Feb 11;111(7):787–94. doi: 10.1007/s00392-022-01986-4 (PMC9242894; doi:10.1007/s00392-022-01986-4)

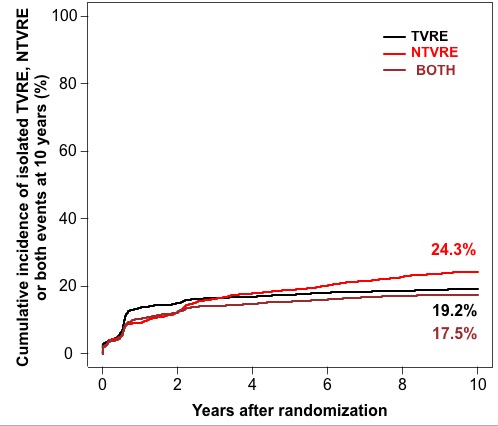

Supplement: Supplementary file 1 — Supplementary file4 (JPG 43 KB) Supplemental Figure 1. Cumulative incidence of target vessel related events, non target vessel related events and both events through 10 years. [file 392_2022_1986_MOESM1_ESM.jpg]

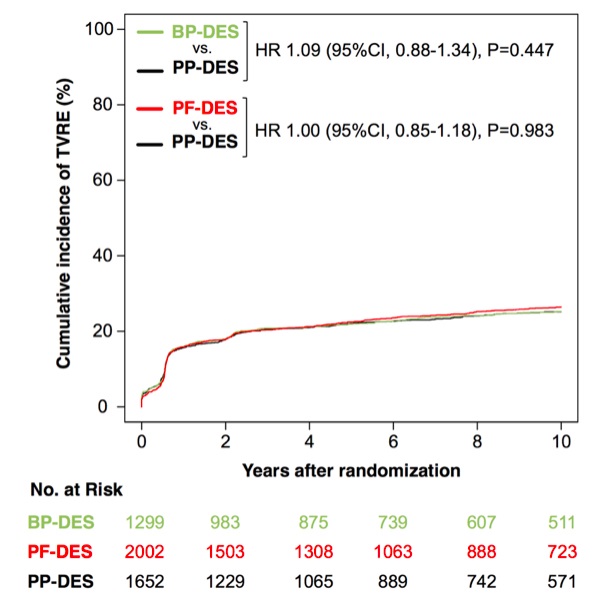

Supplement: Supplementary file 2 — Supplementary file4 (JPG 68 KB) Supplemental Figure 2. Cumulative incidence of target vessel related events based on stent type through 10 years. [file 392_2022_1986_MOESM2_ESM.jpg]

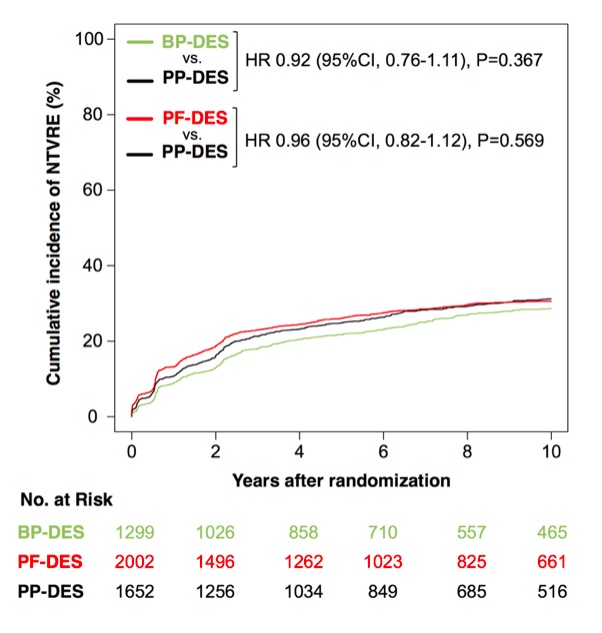

Supplement: Supplementary file 3 — Supplementary file4 (JPG 75 KB) Supplemental Figure 3. Cumulative incidence of non-target vessel related events based on stent type through 10 years. [file 392_2022_1986_MOESM3_ESM.jpg]

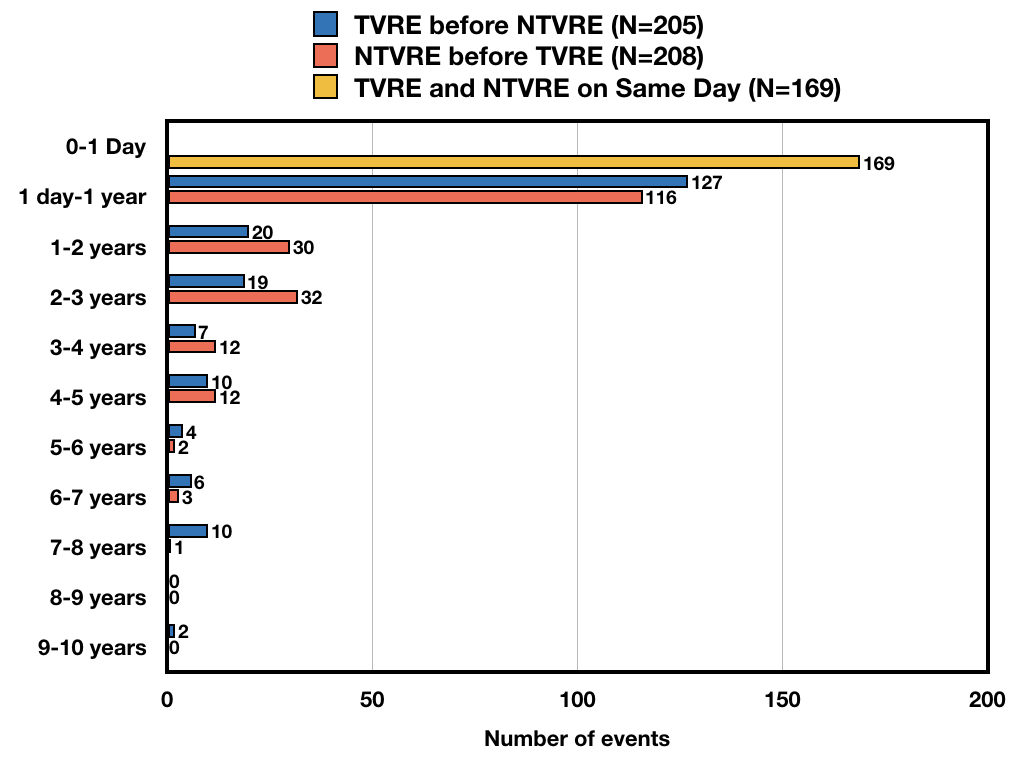

Supplement: Supplementary file 4 — Supplementary file4 (TIFF 3073 KB) Supplemental Fig. 4. Histogram of the time interval between events for patients who experienced both a target vessel and non-target vessel related event through 10 years. Patients could experience a TVRE before a NTVRE, a NTVRE before a TVRE or both events on the same day [file 392_2022_1986_MOESM4_ESM.tiff]
